# Infant formula supplemented with low protein and high carbohydrate alters the intestinal microbiota in neonatal SD rats

**DOI:** 10.1186/s12866-014-0279-2

**Published:** 2014-11-18

**Authors:** Wenguang Fan, Yaru Tang, Yi Qu, Fengbo Cao, Guicheng Huo

**Affiliations:** Key Laboratory of Dairy Science, Ministry of Education, Northeast Agricultural University, Harbin, 150030 China; Hei LongJiang Polytechnic, Harbin, 150111 China

**Keywords:** SD rats, Breast-fed, Low-protein, High-carbohydrate infant formula-fed, Human breast milk-fed, Microbiota

## Abstract

**Background:**

Infant microbiota is influenced by numerous factors, such as delivery mode, environment, prematurity and diet (breast milk or formula) and last but not least, the diet composition. In the diet composition, protein and carbohydrate are very important for the growth of microbiota, many infant fomulas (different ratio protein/carbohydrate) can regulate the development of gut microbiota by different metabolism. The effect of low-protein, high-carbohydrate infant formula on the establishment of microbiota remains unclear, and the effect of human breast milk on the gut microbiota of the rats has also not been reported.

**Results:**

In a 7 d intervention, a total of 36 neonatal SD rats (14 d old) were randomly assigned to the following groups: (1) breast-fed group (A group); (2) low-protein, high-carbohydrate infant formula-fed group (B group); (3) human breast milk-fed group (C group). After 7 days, we selected 6 rats at random from each group to study. Microbial composition in the contents of the large intestines was analysed by Miseq Sequencing. Significantly different (p<0.05) microbial colonisation patterns were observed in the large intestines of breast-fed group from low-protein, high-carbohydrate infant formula-fed and human breast milk-fed rats, but the microbiota of low-protein, high-carbohydrate infant formula-fed group and human breast milk-fed group have high similarity. At the phylum level, the absolute quantity of Bacteroidetes, Firmicutes and Proteobacteria (p<0.001) significantly differentiated in breast-fed group from low- protein, high- carbohydrate infant formula-fed and human breast milk-fed groups. *Lachnospiraceae*, *Bacteroidaceae*, *Porphyromonadaceae* and *Prevotellaceae* were the 4 top families in breast-fed group, but the top 4 families in low-protein, high- carbohydrate infant formula-fed and human breast milk-fed groups were the same, which were *Bacteroidaceae*, *Enterobacteriaceae*, *Porphyromonadaceae* and *Lachnospiraceae*. At the genus level, *Bacteroides* was the most abundant division, their OTUS abundance in three groups was 14.91%, 35.94%, 43.24% respectively.

**Conclusions:**

This study showed that infant formula closer resembling human milk was more different than rats’ breast milk and led to a microbiota profile similar to that for human breast milk-fed neonates. The finding could support a new thinking to develop infant formulas, and provide much more details than what is known previously.

**Electronic supplementary material:**

The online version of this article (doi:10.1186/s12866-014-0279-2) contains supplementary material, which is available to authorized users.

## Background

The gastrointestinal tract is a complex ecosystem that always harbors a diverse bacterial community [1]. During the evolution of both the gut microbiota and the host, the gut microbial community has become an integral component of the host and may affect the host biology [2,3], and it plays a crucial role in health since it is involved in nutrition, pathogenesis, and immunology [4]. Microbial imbalance has been linked to several functional gut disorders. Among its many important functions, the gut microbiota can convert nutritional ingredient into microbial biomass and fermentation end products that can be utilized by the host [5,6].

Infancy is a critical period of colonization of the intestinal microbiota, which affects the adult intestinal microbiota and the future health [7]. The establishment of infant intestinal microbiota is influenced by many factors, the feeding mode does have a crucial impact. Breast-feeding is very important for infants, and breast-fed infants have a gut microbiota that is dominated by *bifidobacteria* [8]. Breast milk is a nutritious food containing the appropriate nutrients for the growing infant, and it can also have a significant impact on the gut microbial composition by virtue of being a source of prebiotics, which beneficially effect the infant by selectively stimulating the growth of one or a limited number of bacteria in the gut [9]. The prevalence of *bifidobacteria* in the gastrointestinal (GI) tract of breast-fed infants has been associated with reduced infection rates [10].

Human breast milk is the optimal nutrition during infancy, but the rates of breastfeeding from one country to another have dropped sharply for practical or medical reasons [11], so the study of infant formulas has been into people's horizons. The infant formula composition has been improved during the last decades to tend to resemble human milk. Cow’s milk protein is one of the major sources of nutrition in formulas but due to difference in the ratio of protein and carbohydrate, the amount of protein per energy content has generally been higher in formula than in human milk to meet the protein and amino acid requirements of infants [12,13]. Numerous babies have been fed high-protein formula for years during the last decades; this infant formula was especially encouraged in at risk populations such as low birth weight babies who had suffered intra-uterine growth restriction to ensure a rapid post-natal catch up growth [14,15], but the current tendency is to reduce protein level in formula for full term healthy babies [16]. A randomized double-blind controlled trial study to evaluate the bifidogenic effect of formula low in protein, allowing a composition closer to that of human milk [17], but the role of low levels of protein as modulators of intestinal microbiota, especially in combination with high levels of carbohydrates, has been poorly investigated.

Understanding the low levels of protein that influence the composition of the microbial community is crucial in regulating the microbiota, which will be need for further research. In this study, we performed multiplex pyrosequencing of the V1-V3 hypervariable regions of 16S rRNA gene with Miseq Sequencing. The aim of this study was to compare the similarity of the gut microbiota of SD rats from three different feeding patterns: breast-fed; low-protein, high-carbohydrate infant formula-fed; human breast milk-fed, and to ascertain how the infant formula low in protein influences early microbial colonisation in the neonatal SD rats’ model.

## Results

A total of 612547 reads and 2998 OTUs were obtained from the 18 samples through pyrosequencing analysis. Each library contains 22473 to 44525 reads, with different phylogenetic OTUs ranging from 93 to 245 (Table 1). We then compared the microbial richness, estimated by the Chao1 and ACE index, and the biodiversity, assessed by a nonparametric Shannon index for the three groups. In our calculations, we took into account the OTU distance unit cutoff of 0.03 (Table 1). We found a high microbial diversity in the infant feces and detected 245 species at most, but the diversity of the infant intestinal microbiota with different feeding patterns was different; the diveristy of fecal microbiota from A group was higher than B and C groups. Using the parametric test for comparisons, we could find significant differences (p <0.001) in richness and biodiversity between the A, B and C samples at the OTU cutoff of 0.03.

**Table 1:** **Richness and diversity indexes relative to each fecal sample (OTU cutoff of 0.03)**

**ID**	**Threshold**	**Number of reads**	**Number of OTUs**	**Alpha diversity**			
				**ACE**	**Chao1**	**Shannon**	**Simpson**
A1	0.03	22473	235	266	263	3.87	0.0514
A2	0.03	32037	221	253	263	3.68	0.0466
A3	0.03	33278	245	261	258	3.55	0.0804
A4	0.03	39229	245	308	286	3.9	0.0392
A5	0.03	26630	223	257	254	3.62	0.062
A6	0.03	31644	161	193	202	3.23	0.0742
B1	0.03	27296	178	215	233	2.67	0.1518
B2	0.03	39824	143	218	192	1.85	0.3823
B3	0.03	35116	138	156	155	2.97	0.0862
B4	0.03	29869	153	173	176	2.45	0.2063
B5	0.03	37604	136	158	161	1.95	0.2763
B6	0.03	34103	170	200	221	2.73	0.1307
C1	0.03	44525	135	166	176	2.69	0.1097
C2	0.03	31091	134	172	165	2.2	0.2155
C3	0.03	37837	139	185	195	1.86	0.2737
C4	0.03	25521	107	140	148	2.08	0.2094
C5	0.03	41564	142	169	169	2.1	0.2695
C6	0.03	42906	93	111	108	1.87	0.291
***P*** **value**		**0.227**	**0.0000127**	**0.00018**	**0.000219**	**3.58E-06**	**0.002472**

(p<0.05 indicate significant differences).

**Figure 1:**
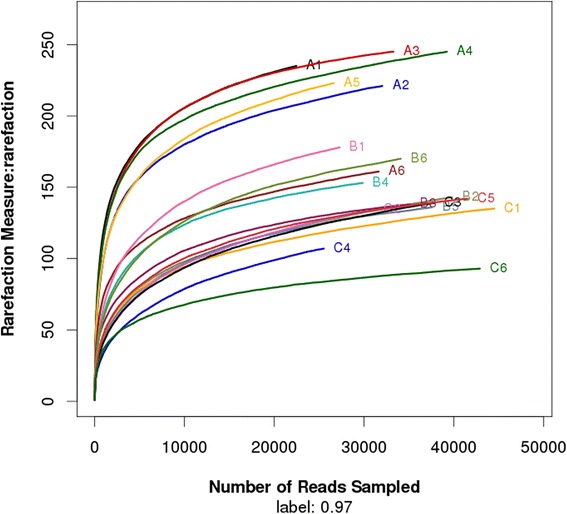
**Rarefaction analysis of the different samples.** Rarefaction curves of OTUs clustered at 97% phylotype similarity level.

The rarefaction curves tend to approach the saturation plateau (Figure 1). The Good’s coverage index reveals that 92% to 96% of the species were obtained in eighteen samples. The samples from A group (e.g., the samples of A1, A2, A3, A4 and A5 except A6) were plotted in the upper part .This rarefaction curve indicated a large variation in the total number of OTUs in different samples, but the sequence coverage was still sufficient to capture the diversity of the bacterial communities, whereas the OTUs density was larger in the upper layer than the lower parts. The samples from B and C groups were plotted in the lower parts. The same tendency was found in species accumulation curves and Shannon-Wiener curves, it tends to approach the saturation plateau, which meant that the database of 16S rRNA gene sequences was very abundant, that reflected the vast major of microbial information (see Additional file 1 and Additional file 2).

### Taxonomic composition

**Figure 2:**
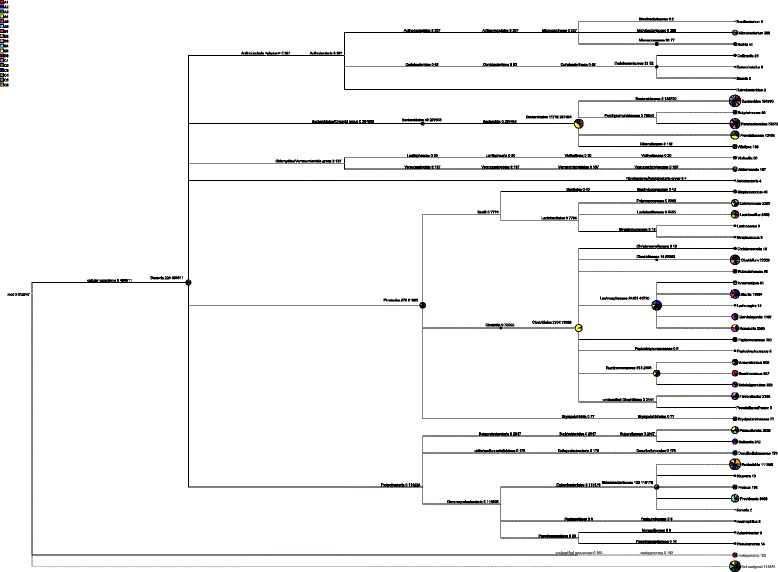
**Representative bacterial community composition in large intestine of different vegetation classes.** Shown are the percentages of the classified sequences. Representative pyrosequencing reads were selected from each group of the OTUs clustered within the 3% genetic distance by the MOTHUR program.

The eighteen samples comprised of different numbers of OTUs and OTU abundances. All sequences were classified from phylum to genus according to the program Mothur using the default setting. Sequences that could not be classified into any known group are assigned as no rank. These bacterial OTUs can be assigned into 11 different phyla, 44 families or 78 genera (Figure 2).

**Figure 3:**
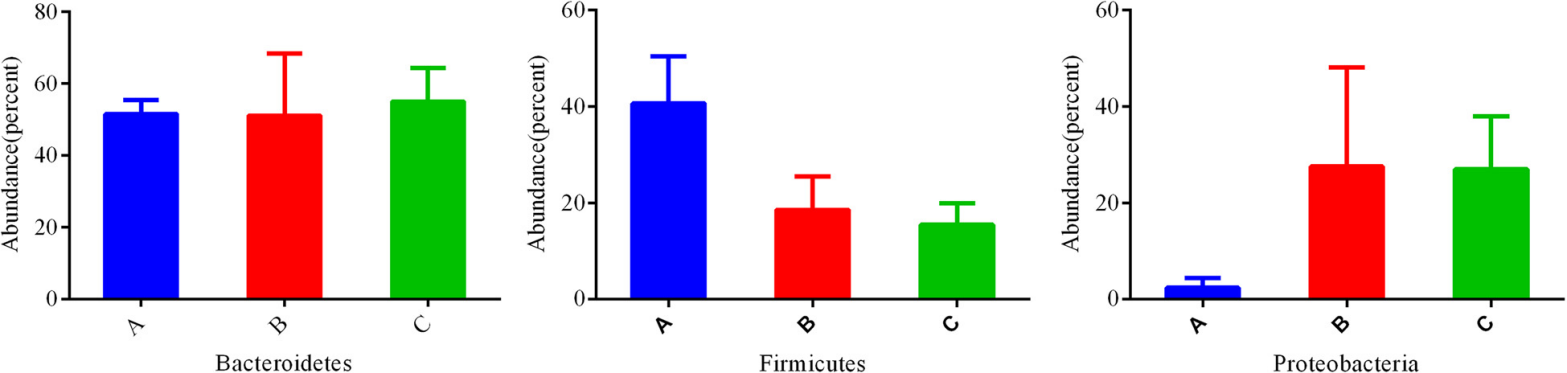
**Relative abundance of the intestinal flora at the phylum level in infants (percent).** The mean abundance of the intestinal flora at the phylum level in breastfed pups **(A)**. The mean abundance of the intestinal flora at the phylum level in weaned pups fed on formula **(B)**. The mean abundance of the intestinal flora at the phylum level in weaned pups fed on human breast milk **(C)**.

The representative sequences at phylum level were listed in Figure 3. Bacteroidetes, Firmicutes, Proteobacteria were common to the whole 18 libraries, which comprised 94.52%, 97.11%, 97.53% of the total reads in the libraries of A, B and C groups, respectively. Bacteroidetes was the most abundant division, comprising approximately from 18.79% to 66.75% reads across all samples, their mean OTUs abundance in A, B and C groups was 51.07%, 49.35%, 55.97% respectively. Firmicutes was the secondary bacterial phylum in A group (41.01%), but its abundance in B and C groups was 18.11% and 15.77%. The secondary bacterial phylum in B and C groups was Proteobacteria, its abundance was 29.40% and 25.67% respectively. Statistical analysis indicated that the absolute quantity of Bacteroidetes, Firmicutes and Proteobacteria (p<0.001) significantly differentiated A from B group, and they also differentiated A from C group (p<0.001). The average reads of No_rank group in A, B and C groups was 64, 57 and 105 reads.

**Figure 4:**
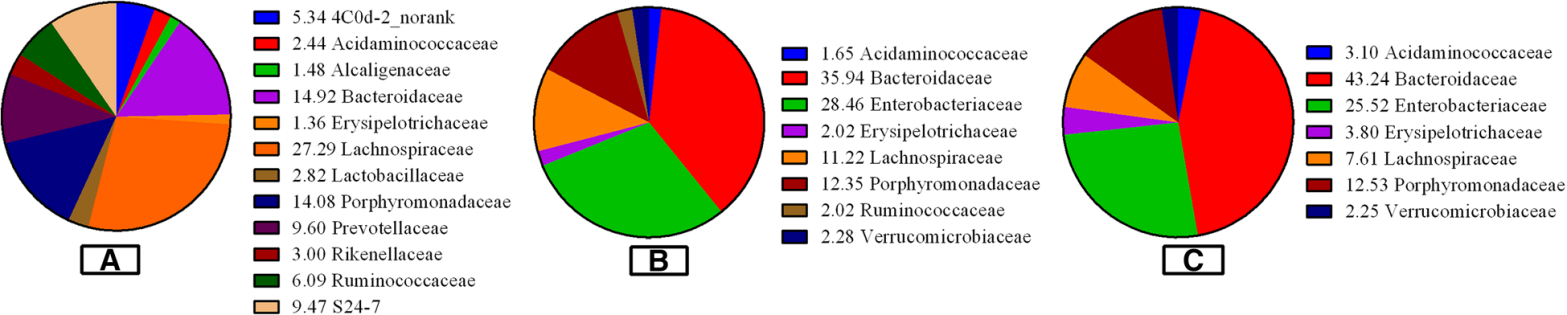
**Relative abundance (>1%) of the intestinal flora at the family level in infants (percent).** The mean abundance of the intestinal flora at the family level in breastfed pups **(A).** The mean abundance of the intestinal flora at the family level in weaned pups fed on formula **(B)**. The mean abundance of the intestinal flora at the family level in weaned pups fed on human breast milk **(C)**.

At family level, there were 6 shared families (abundance > 1%) among the total 44 families exist in all samples (Figure 4), namely, *Acidaminococcaceae*, *Bacteroidaceae*, *Enterobacteriaceae*, *Erysipelotrichaceae*, *Lachnospiraceae*, and *Porphyromonadaceae*. The top 4 families in group A, by descending read abundance, were *Lachnospiraceae* (27.29%), *Bacteroidaceae* (14.91%), *Porphyromonadaceae* (14.08%) and *Prevotellaceae* (9.60%) respectively. The top 4 families in B and C groups were same, which were *Bacteroidaceae* (35.94% versus 43.24%), *Enterobacteriaceae* (28.46% versus 25.52%), *Porphyromonadaceae* (12.35% versus12.53%) and *Lachnospiraceae* (11.22% versus 7.61%),whereas the fifth family in B and C groups was *Verrucomicrobiaceae* (2.28%) and *Erysipelotrichaceae* (3.80%). From the Figure 4, it was obvious that the gut microbiota of B between C groups at the family level had high similarity.

**Figure 5:**
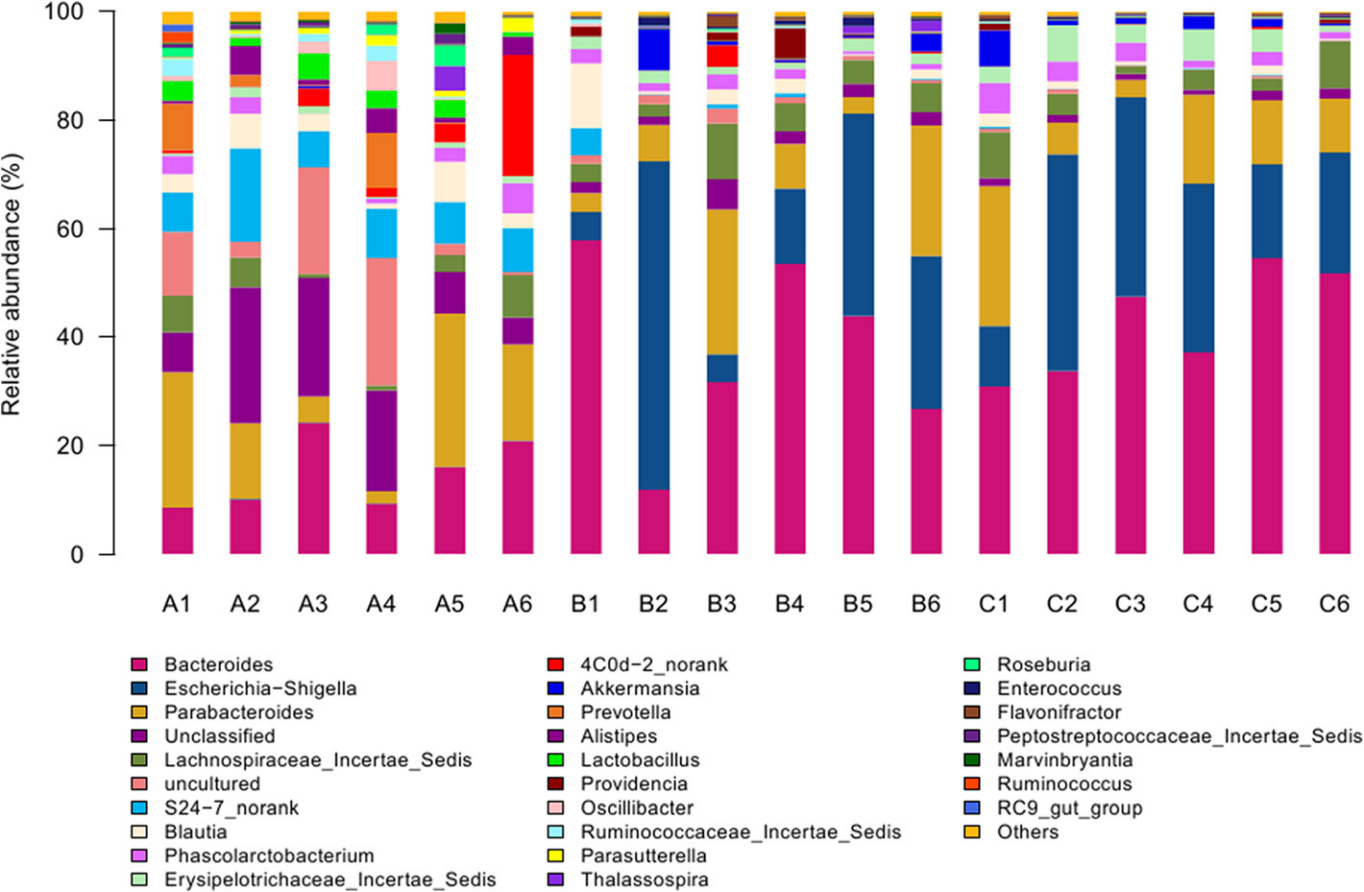
**Bacterial composition of the different communities at the genus level.** Relative read abundance of different bacterial genus within the different communities. Sequences that could not be classified into any known group were assigned as No_Rank.

Detected OTUs were distributed among 78 different bacterial genera. *Bacteroides* was the most abundant division (Figure 5), comprising approximately from 1909 to 22073 reads across all samples, their mean OTUs abundance in A, B and C groups was 14.91%, 35.94%, 43.24% respectively, and it was the most abundant genus in B and C groups, but unclassified genus was the most abundant species in A group, its mean abundance was 15.04%. The secondary genus in B and C groups was *Escherichia-Shigella*; its mean abundance was 27.06% and 25.06%. The third genus in B and C groups was *Parabacteroides*, its mean abundance was 12.22% and 12.49%, interestingly, this genus in A group was the same place. *Lactobacillus* was one of the most famous probiotics, its mean abundance was 2.82% in A group, but it was not easy to be find in B and C groups. The mean abundance of S24-7_norank genus in A group was 9.47%, which was 10 fold in B group, and 90 fold in C group.

### Bacterial community variation

**Figure 6:**
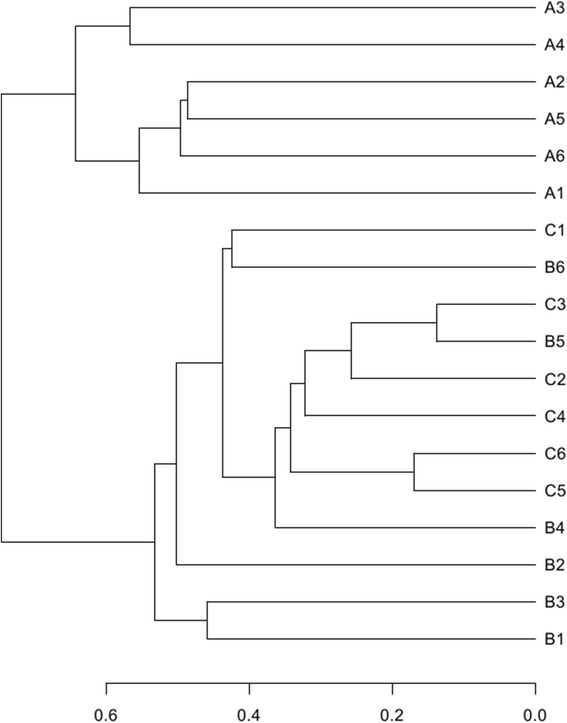
**Multiple samples similarity tree.**

**Figure 7:**
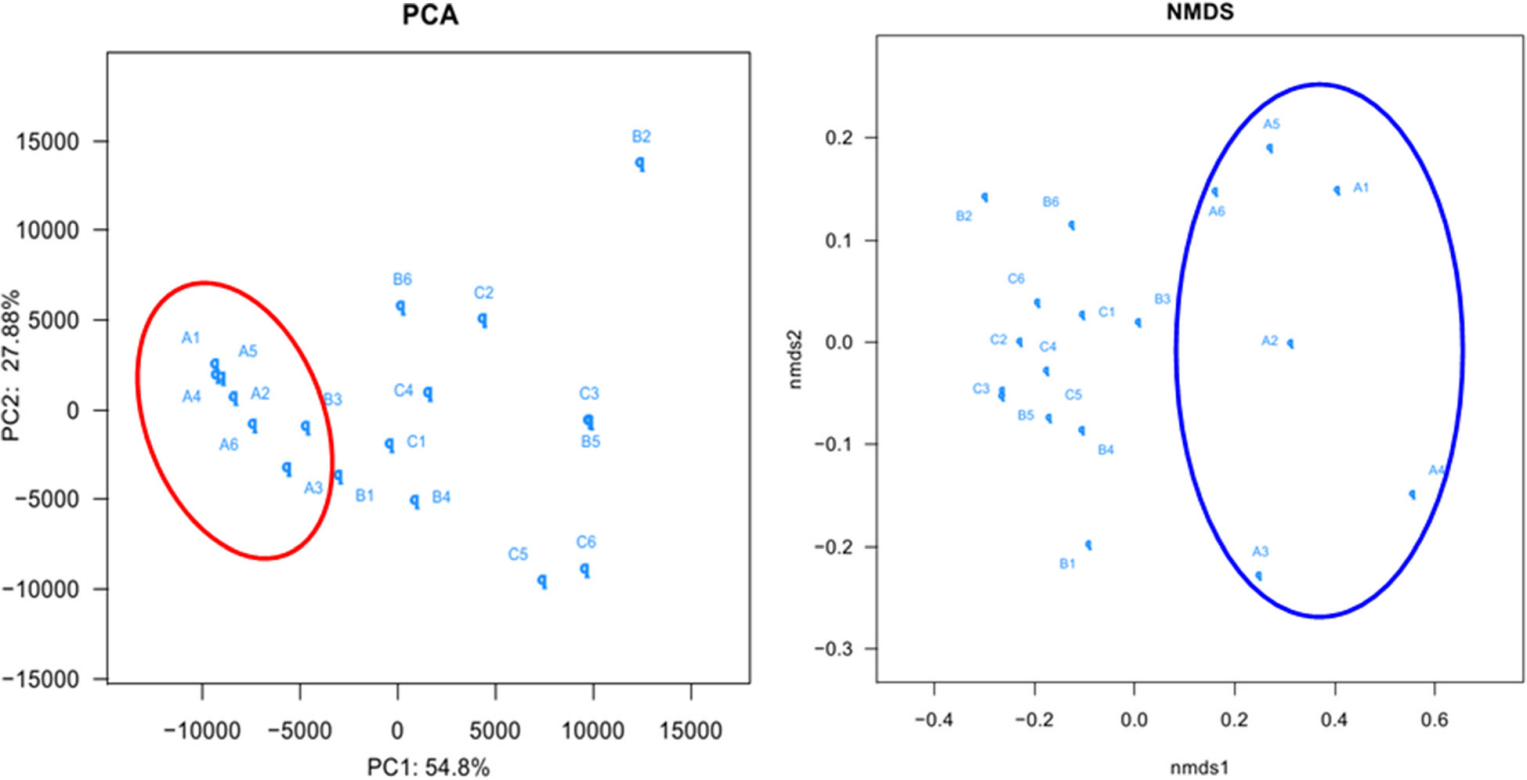
**Sample sorting analysis.** Scatter plot of PCA-score showing similarity of the 18 bacterial communities based on Unifrac distance. Principal components (PCs) 1 and 2 explained 54.8% and 27.88% of the variance, respectively. NMDs showing the difference of bacterial communities according to Bray-Curtis distance.

**Figure 8:**
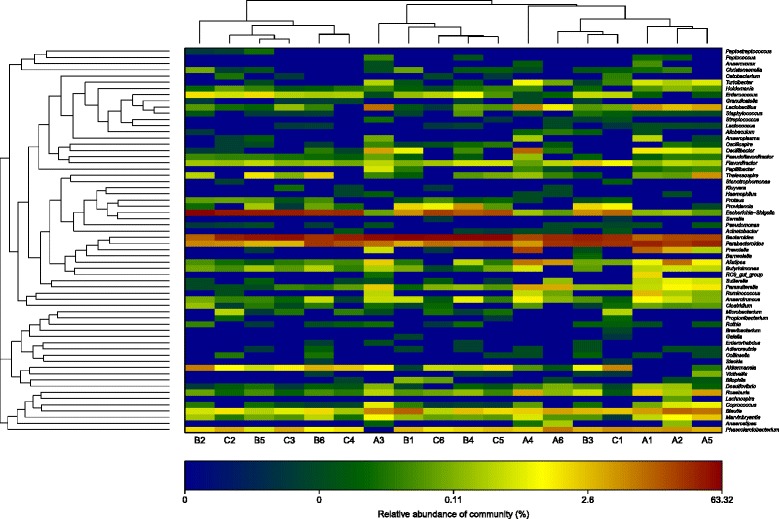
**Bacterial distribution of the top 100 abundant genus among eighteen samples.** Double hierarchical dendrogram shows the bacterial distribution. The heatmap plot depicts the relative percentage of each bacterial genus within each sample. The relative values for bacterial family are indicated by color intensity with the legend indicated at the top right corner.

**Figure 9:**
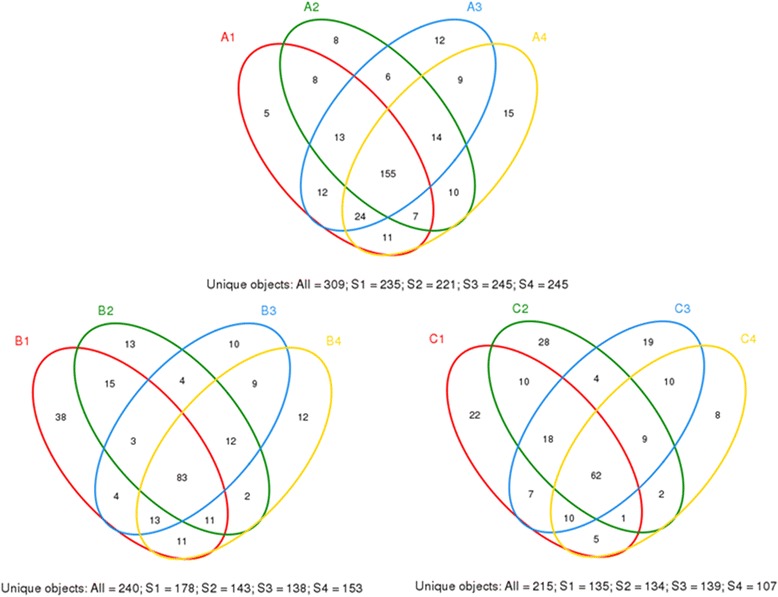
**Venn diagram showing the unique and shared OTUs (3% distance level).**

The hierarchical cluster analysis (Figure 6) using MVSP 3.1 software showed that the B and C communities grouped together, and then clustered with the A communities in order. The principal component analysis with the weighted UniFrac distance and heatmap analysis were determined using pyrosequencing data to corroborate further the findings from these DNA fingerprinting methods. The principal component analysis (PCA) score plot revealed that the A communities harbored characteristic bacterial communities, and the entire A samples grouped to the left of the graph along PC1, which accounted for 54.8% of the total variations. The B and C samples were closely related, whereas the B2 sample was separate from the other samples along PC2, which represented 27.88% of the total variations (Figure 7). Overall, the two PCA axes explained 82.68% of the variation between the different communities. The NMDS analysis based on the Bray-Curtis distance, also confirmed bacterial communities in A group were more similar than the B and C groups (Figure 7). The direction and position of the environmental factors, calculated with the envfit function in the R vegan package [18], suggested the bacterial community in the SD rats’ gut micro biota was somewhat related to the feeding mode. However, the correlation did not reach statistical significance. Species rank abundance distribution curves (see Additional file 3) revealed that the OTUs present in five libraries (A1-A5) contained the most abundant OTUs in any library, whereas the OTUs observed in other libraries seemed to be relatively low in diversity. The hierarchical heatmap was based on abundant bacterial community at genus level, which generally indicates three groups (Figure 8). One was mainly composed of A4, A6, B3, C1, A1, A2 and A5 samples; the other group was chiefly clustered by other samples (B2, C2, B5, C3, B6, C4, A3, B1, C6, B4 and C5). According to the heat map, high similarity of the communities could be found in the samples from A group, and relatively high similarity was also found in B and C groups. The species shared among these communities were determined via a Venn diagram to compare the relationships among these communities in detail. The result showed that the number of species shared in A (A1-A4), B (B1-B4) and C (C1-C4) communities was 155, 83 and 62 respectively, which showed A group had higher number of shared species (Figure 9).

## Discussion

Infant complex intestinal microbial communities are believed to provide some benefits to their host [5]. The course of microbial colonization of the newborn gut is determined by a complex interaction of factors, in addition to genetics, dietary habits, and sedentary life styles, altered nutritional experiences in early life via malprogramming effects in target organs, and also contribute to the ecology of the gut microbiota. In several of feeding patterns, the breast-fed is the best feeding mode. The ability of breast milk to modulate the composition of the infant gut microbiota contributes to its beneficial health effects. Among the properties of breast milk affecting the development of the infant gut microbiota are the type and quantity of protein and carbohydrate [19]. The rate of breast fed has been declining; many types of infant formulas have now received increasing attention. Diets that are high in protein but reduced in carbohydrate contents provide a common approach for achieving weight loss, but long-term adherence to such diets may increase risk of colonic disease [20]. HP diet consumption should be considered with some caution because of its ability to modify the composition of the luminal content and the morphology and metabolic oxidative capacity of colonic epithelial cells in rats [21]. In the studies of weaned piglets, fermentation of high protein often coincided with the growth of potential pathogens, thereby increasing the risk for infectious diseases. In the meantime, fermentation of dietary protein could produce detrimental substances, including ammonia, amines and phenols, these substances have been associated with various detrimental effects for the host animal [22]. Reducing dietary crude protein (CP) level, as a strategy to solve the problems, has been found to limit the frequency and the severity of digestive problems in piglets [23]. At the same time, reducing the amount of dietary protein has also been proved as an alternative option to avoid excessive protein fermentation [24], and this approach potentially contributed to improvements in health conditions and growth performance [25]. Another study has been shown that reducing dietary intake of protein could improve gut function after weaning [26]. Low protein formulas have begun to be used in neonates, its effect allowing a composition closer to that of human milk [17]. Apart from the low protein, the high carbohydrate diet have received more and more attention, one study have been investigating the long-term consequences of altered postnatal nutrition independent of fetal effects by artificially rearing neonatal rat pups on a high-carbohydrate (HC) milk formula during the suckling period, they found high-carbohydrate pups correlates with several adaptations in pancreatic β cells including the autonomic regulation of insulin secretion, and the source of calories varied (from fat-rich in rat milk or the high-fat (HF) milk formula to high carbohydrate in the HC milk formula) within the context of an isocaloric and isonitrogenous milk formula [27,28]. In the context, the effect of high protein infant formula did not allow a composition closer to the human milk and may lead to some risk, we did not design a group of rats fed with high protein infant formula. In the study, we performed multiplex pyrosequencing of the V1-V3 hypervariable regions of 16S rRNA gene of the SD rats’ gut microbiota with Miseq Sequencing, the SD rats from three different feeding patterns: breast-fed; low-protein, high-carbohydrate infant formula-fed; human breast milk-fed, and to compare the similarity of the gut microbiota of the SD rats from different feeding patterns, and our results could provided a theoretical and technical support for future studies of the infant formulas.

The major finding of our study was that feeding rat neonates a low protein with high carbohydrate formula induced dramatic changes in the developmental profile of SD rats’ intestinal microbiota. Indeed feeding neonate rats LP (low-protein) formula and human breast milk both decreased bacterial abundance and diversity in the large intestine, while the bacterial abundance and diversity in the large intestine of low- protein with high-carbohydrate infant formula-fed rats between human breast milk-fed rats was similar. Bacteroidetes, Firmicutes, Proteobacteria occupied 94.52%, 97.11%, 97.53% of the total reads in the libraries of A, B and C groups, respectively, which were the dominating gut microbiota, in agreement with the previous study describing such phyla as those contributing to the majority of infant gut microbiota [29], but the absolute quantity of Bacteroidetes, Firmicutes and Proteobacteria (p <0.001) significantly differentiated A from B and C groups. The abundance of Bacteroidetes was the highest in three groups, Proteobacteria was the most secondary abundant, followed by Firmicutes in B and C groups, which was inconsistent with the previous study [30], the incongruence may probably be due to the differences in host and the host living conditions. Surprisingly, the phylum generally made up a very small proportion of bacterial sequences retrieved from rats’ intestine, which was agree with the Claesson’s study [31]. There were 6 shared families (abundance > 1%) among the total 44 families exist in all samples at family level. The most abundant microbiota at the family level was the *Lachnospiraceae* in A group, but the most abundant microbiota at the family level was the *Bacteroidaceae* B and C groups. Interestingly, the abundance, diversity and species (abundance > 1%) of B and C group almost the same from Figure 4. At the genus level, *Bacteroides* was the most abundant division in three groups. Probiotics have been widely used; they may prevent pathogens from proliferating in the intestinal tract, and in the culture environment and may improve condition by securing optimal use of the feed, or stimulating the immune system of the host [32]. As one of the most famous probiotics, the mean abundance of *Lactobacillus* was 2.82% in A group, but it was not easy to be found in B group. The reason may be the breast milk have a strong prebiotic effect for the neonate’s developing microbiota [33], and it may supply directly this microbiota [34]. Surprisingly, *Lactobacillus* was not also found in the human breast milk-fed group, which may probably be due to the different physiological structure and function between rats and human, and the host living conditions. Actinobacteria were prevalent members of the intestinal bacterial communities and they were more abundant in the study, which are widely distributed in both terrestrial ecosystems, and they played a crucial role in the recycling of refractory biomaterials through decomposition and humus formation, the production of their secondary metabolites were potent antibiotics [35], but in the study, there was a strong DNA extraction bias which was evident by absence of Actinobacteria (including *Bifidobacterium* spp.) in the analysed samples, differ considerably from many previous studies, they have reported that the microbiota of breast-fed infants was dominated by *Bifidobacteria* [36,37], but it was consistent with the previous study [38], it showed that *Bifidobacteria* were not appear at a specific period after birth, and thereafter persisted as a minority population, and they also pointed out *Bifidobacteria* in studies of the infant GI microbiota may be excessive emphasis on its role to health. Although such technical error caused some shift in the detected microbial community, we thought the results presented here suggest numerous future avenues of research.

Intestinal microbiota has been widely recognized in the context of mammalian hosts [39]. In the present study, PCA and heatmap plots of the bacterial communities derived from SD neonate rats, and the 16S rRNA gene fingerprinting based analyses, suggested that the rats harbored different intestinal microbiota from A group from B and C groups, and showed that the gut bacterial communities were more similar to the B and C groups than the A group. Species rank abundance distribution curves indicated that A group also contained the most abundant OTUs in any library, whereas the OTUs observed in other libraries tended to be relatively low in abundance. In addition, the Venn diagram indicated that A group shared more species than the B and C libraries, each group had core intestinal microbiota. These results indicated that feed might significantly influence the composition of the gut microbiota.

## Conclusion

Although it is now apparent that the feeding pattern is not the sole determinant of the levels of bacteria in the infant gut, it is clear that feeding does have a crucial impact. Rats from formula and human milk group suffered substantial stress, were forced fed, had no contact with adult rats, and were maintained in the same living circumstance, and the level of protein with high carbohydrate in formulas did have immediate consequences on gut ecology, we concluded the composition of the formula may be the factor causing similar microbiota profiles. The study provided a more comprehensive understanding of the structure and diversity of the intestinal microbiota diversity in three different feeding modes. Furthermore, such low protein formula-fed also had high similar bacterial abundance and diversity with human breast milk fed rats, it could provide us a new idea for future studies of the infant formulas.

## Methods

### Animals

A total of 36 pups, derived from a breeding colony of SD rats supplied by Vital River Laboratory Animal Technology Co. Ltd., were used in this study. The progenitor mice were 8 weeks of age and were acclimatized for 30 days prior to breeding. All the rats were determined to be healthy on the basis of individual physical examinations and pathogen free based on results of the routine microbiological screening performed in the colony. All the rats were maintained in stainless steel Eurostandard Type II cages (36.5 × 20.7 × 14.0 cm) protected with filter tops. The cages had solid bottoms, were covered with Aspen chip bedding and were provided with some nesting material. The environment in the room consisted of a temperature range of 23°C (±3°C), a relative humidity of 55 ± 15% and an artificial illumination of a 12-h light/dark cycle. They were maintained on a standard laboratory diet and were allowed free access to water.

Day of birth was referred to as Day 0 of neonatal life. At the commencement of the study, all the pups were 14 days of age, and their mean body weight was 33.27 ± 1.01 g. Throughout the study period, all the pups in the breastfed group (A group) had free access to the dam's nipples, the pups in formula-fed group (B group) had free access to a standard moist diet (Table 2) consisting of a porridge made by adding warm water to an infant formula at a final proportion of 65 g of infant formula/15 mL of water, the pups in C group had free access to human breast milk, which was bought in Harbin city maternal and child health care center, Heilongjiang province. The B and C groups were administered with 1.0-mL diet every 4 h, including nighttime, using sterile 1.5-mL syringes attached to specialized feeding needles before killing. Each group had a separate feeding needle. After each feeding, the needles were washed, resterilized, and stored in labeled, sealed, sterile conical tubes until the next feeding time. The 1.5-mLsyringes were discarded after each feeding. The pups were weighed and measured daily for weight loss or gain. All the adult rats were fed a standard mouse chow adlibitum. Tap water was provided without restrictions to adult and infant mice in polycarbonate bottles. The animals were weighed daily during the intervention study and handling was done in the same time range to avoid the influence of biological rhythms.

**Table 2:** **Nutrient content of the formula**

**Nutrient**	**Standard**	**Nutrient**	**Standard**
Energy (kcal)	100	Iron (mg)	1.15
Protein (g)	1.8	Zinc (mg)	1.2
α-lactoalbumin (g)	1.08	Copper (μg)	38
L-carnitine(mg)	14	Iodine (μg)	16
Arachidonate (μg)	20	Selenium (μg)	6
Aocosahexaenoic acid (μg)	1	Vitamin A (μg)	116
Linoleic acid (mg)	900	Vitamin D (μg)	1.7
α-linoleic acid (mg)	90	Vitamin E (IU)	2.6
Carbohydrate (g)	10.2	Vitamin K (μg)	15
Galactooligosaccharide (g)	0.14	Thiamine (μg)	174
Fructo-oligosaccharide (g)	0.05	Riboflavin (μg)	280
Fat (g)	5.37	Niacin (μg)	870
Calcium (mg)	117	Pantothenic acid (μg)	1062
Phosphorus (mg)	75	Pyridoxin, vitamin B-6 (μg)	108
Sodium (mg)	49.5	Cobalamin, vitamin B-12(μg)	0.38
Potassium (mg)	105	Folic acid (μg)	29
Chloride (mg)	145	Vitamin C (mg)	19.7
Magnesium (mg)	6.25	Choline (mg)	21.2
Taurine (mg)	1.38	Biotin(mg)	3.3
Myo-inositol (mg)	20		

Note: All nutrients are expressed per 100 kcal except Energy.

This study was performed at the Northeast Agricultural University. Pilot experiments were performed to optimize all the experimental procedures including handling and treatments. The experimental protocol was approved by the Institutional Animal Care and Use Committee of Northeast Agricultural University under the approved protocol number SRM-06.

### Study design

**Figure 10:**
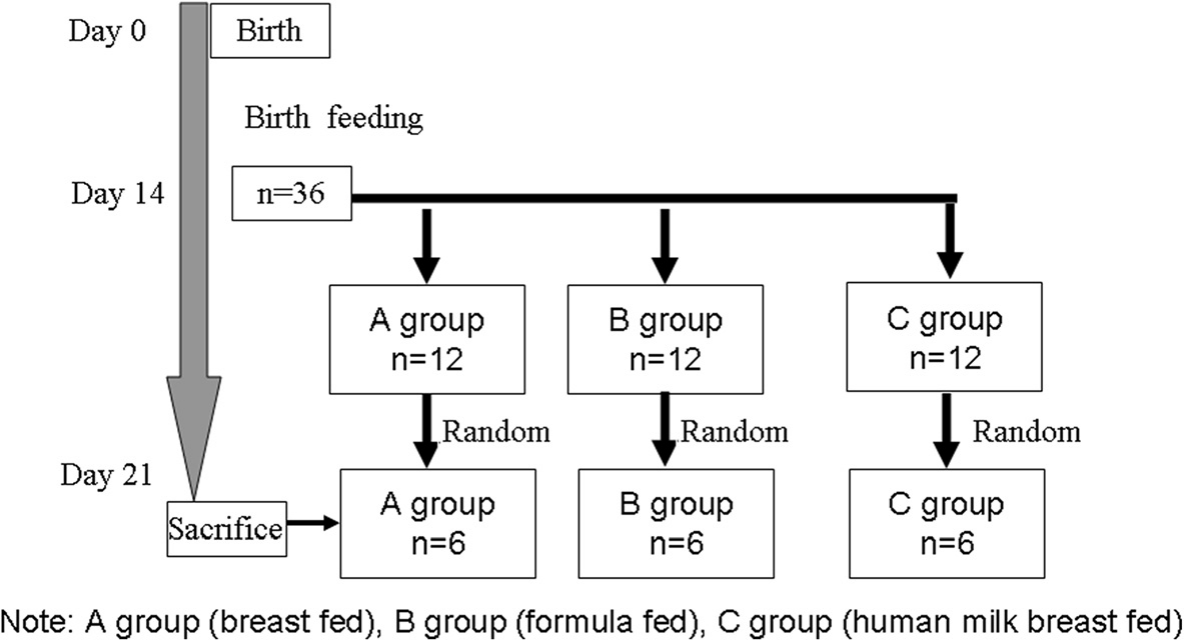
**Experimental study design.**

The animals were randomly assigned to dietary intervention groups and treated as described in Figure 10. Intervention was given for 7 days. The study groups were breastfed (unweaned) pups (A group, n = 12); weaned pups fed on formula (B group, n = 12); and weaned pups fed on human breast milk (C group, n = 12). After 7 days, we selected 6 rats at random from each group to study.

### Fecal sample collection and DNA extraction

After the 7-day diet intervention, the animals were anesthetized with isoflurane and samples were collected. Immediately after euthanasia by cervical dislocation, the entire intestinal tract was removed from the pups on Day 21. The full contents of the large intestines were emptied, and were placed into sterile polypropylene centrifuge tubes and stored provisionally in a portable refrigerator at-20°C, and transferred to laboratory within 24 hours and kept frozen at −80°C until DNA extraction. DNA was extracted according to the protocol described by the E.Z.N.A Soil DNA Kit (OMEGA).

### PCR amplification and pyrosequencing

A region, 506 bp in the 16S rRNA gene, covering the V1-V3 region was selected to construct community library through tag pyrosequencing. The bar-coded broadly conserved primers 27 F and 533R containing the A and B sequencing adaptors were used to amplify this region. Universal bacterial primer set 27 (5’-3’ AGAGTTTGATCCTGGCTCAG) and 533R (5’-3’ TTACCGCGGCTGCTGGCAC) covering V1-V3 regions of SSU were synthesized by Shanghai Majorbio Bio-pharm Technology Co., Ltd. (Majorbio as below). Different barcode sequences were added at the 5’ end of the forward primer for multiplexed pyrosequencing. PCR were carried out in a 20 μL reaction volumes containing 10 ng DNA template, 2 μL dNTPs (2.5 mM), 0.4 μL of each primer (5 μΜ) and 0.4 UμL FastPfu Polymerase (Applied Biosystems) in the appropriate 56 FastPfu Buffer (4 μL) and de-ionized ultrapure water (to 20 μL). The protocol was optimized with low cycles for better accuracy and reliability of the subsequent data analysis. The PCR condition were initial denaturation at 95°C for 2 min, followed by 25 cycles of denaturation at 95°C for 30 s, annealing at 55°C for 30 s and extension at 72°C for 45 s, with a final extension phase at 72°C for 10 min. PCR products (3 μL) were checked on a 2% agarose gel. PCR products were purified using MiniElute PCR purification kit (AXYGEN) and quantified using the ABI GeneAmp® 9700 system. Samples were then pooled at equal concentrations. Parallel tagged sequencing was performed using a Miseq Sequencing in Majorbio.

### Bioinformatic analysis

Data preprocessing was performed mainly upon software of mothur [40]. These sequences were clustered to OTUs (operational taxonomic units) at 97% sequence identity by using mothur (furthest neighbor method) and chopseq (Majorbio). Rarefaction analysis was performed by mothur and plot-rarefaction (Majorbio). From these, the Shannon diversities and the Chao1 richness estimations were calculated by mothur. The weighted UniFrac distance was used to quantify differences in community composition. Heatmap figure and Venn diagrams were implemented by R packages heatmap [41] and Venn diagram [42], respectively. In addition, weighted principal component analysis (PCA) and Nonmetric Multidimensional Scaling (NMDS) diagrams were generated by using R package vegan [18] to demonstrate the clustering of different samples.

### Statistical analyses

Differences between populations had been analyzed using parametric (ANOVA) and nonparametric statistical methods. All results are presented as the mean value (± SE). Differences between groups were declared significant at p <0. 05.

### Availability of supporting data

16S rRNA gene sequences supporting the results of this article are available in the GenBank Database (https://www.ncbi.nlm.nih.gov/genbank/) under the accession number SRP048909.

## Additional files

Additional files 1: Figure S1. Shannon Wiener curves of samples. Additional files 2: Figure S2. Species accumulation curves. Additional files 3: Figure S3. OTU Rank-Abundance curves.

## Abbreviations

ANOVA: Analysis of variance
DNA: Deoxyribonucleic acid
PCR: Polymerase chain reaction
SE: Standard error
PCA: Principal component analysis
