# Supplementary figures and images for: Infant formula supplemented with low protein and high carbohydrate alters the intestinal microbiota in neonatal SD rats
Source: BMC Microbiol. 2014 Nov 18;14:279. doi: 10.1186/s12866-014-0279-2 (PMC4243196; doi:10.1186/s12866-014-0279-2)

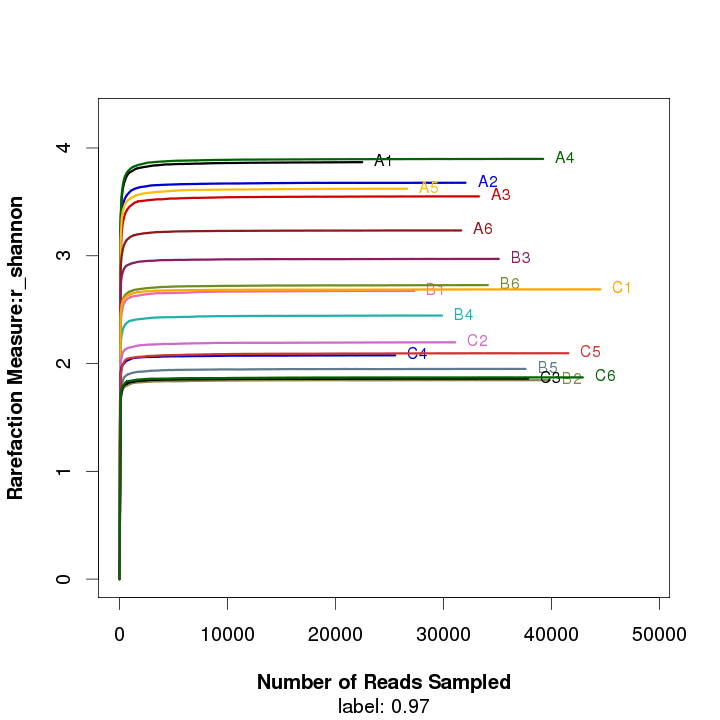

Supplement: Additional files 1: Figure S1. — Shannon Wiener curves of samples. [file 12866_2014_279_MOESM1_ESM.tiff]

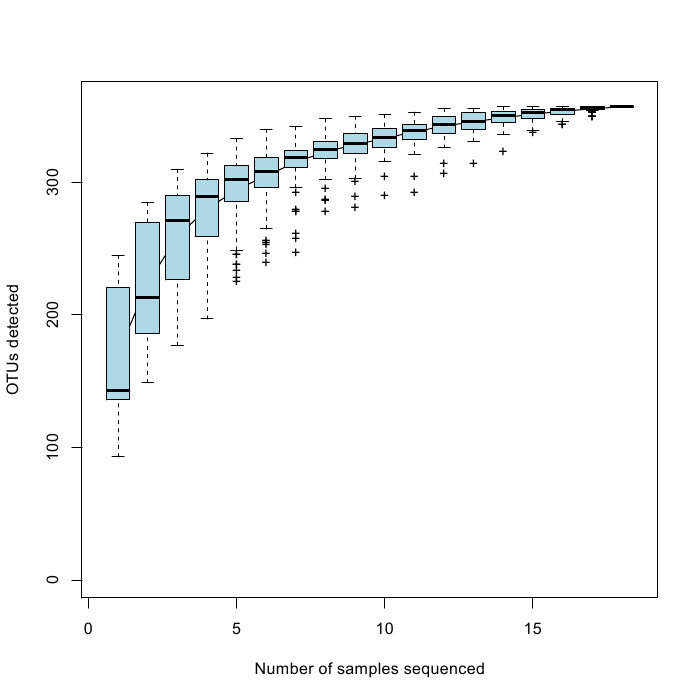

Supplement: Additional files 2: Figure S2. — Species accumulation curves. [file 12866_2014_279_MOESM2_ESM.tiff]

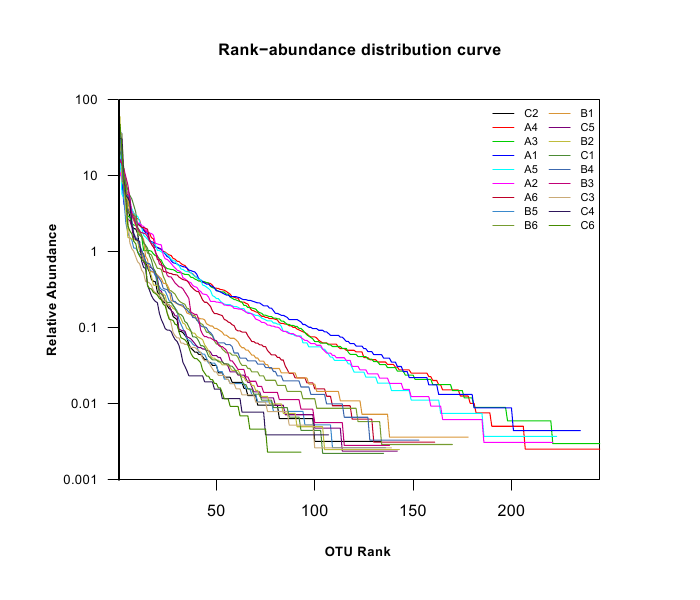

Supplement: Additional files 3: Figure S3. — OTU Rank-Abundance curves. [file 12866_2014_279_MOESM3_ESM.tiff]
